# 

**Published:** 1842-10

**Authors:** 


### BOOKS RECEIVED FOR REVIEW.

BRITISH.

1. Elements of General Pathology. By the late John Fletcher, M.D. Edited by J. J. Drysdale, M.D., and J. R. Russel, M.D. Edinb. 1842. 8vo, pp. 519. 10s. 6d.

2. The Elements of Materia Medica and Therapeutics. By J. Pereira, M.D. F.R.S. &c. &c. Second Edition, enlarged and improved.—London, 1842. Two Vols. 8vo, pp. 1926. 45s.

3. On the Comparative Advantages of Lithotomy and Lithotrity, and on the circumstances under which one method should be preferred to the other. The Jacksonian Prize Essay for 1838. By Edwin Lee, M.R.C.S.—Lond. 1842. 8vo, pp. 30. 2s. 6d.

4. The Nervous System and its Functions. By Herbert Mayo, F.R.S.—London, 1842. 8vo, pp. 182.

5. Dr. Hooper's Physician's Vade-Mecum. New Edition, considerably enlarged and improved, with an Outline of General Pathology and Therapeutics. By W. A. Guy, M.B. Cantab.—London, 1842. 8vo, pp. 493. 10s.

6. The Anatomist's Vade-Mecum; a System of Human Anatomy. By Erasmus Wilson. Second Edition.—London, 1842. 8vo, pp. 595. 12s. 6d.

7. The Practice of Medicine; or a Treatise on Special Pathology and Therapeutics. By R. Dunglison, M.D.—Philadelphia, 1842. Two Vols. 8vo, pp. 572, 750.

8. A Description of the Mineral Springs of Aix-la-Chapelle and Borcette. By L. Wetzlar, M.D., Physician at Aix-la-Chapelle.—London, 1842. 8vo, pp. 88. 2s. 6d.

9. An Introductory Lecture to the Course of Institutes of Medicine, &c. delivered in Jefferson Medical College, Nov. 1, 1841. By R. Dunglison, M.D.—Philadelphia, 1841. 8vo, pp. 24.

10. Discourse on the Enlarged and Pendulous Abdomen, &c. By R. Frankum, Esq. Surgeon. Second Edition.—London, 1842. 8vo, pp. 121. 5s.

11. Observations on the Admission of Medical Pupils to the Wards of Bethlem Hospital, for the purpose of studying Mental Diseases. 2d Edition. By John Webster, M.D.—London, 1842. 8vo, pp. 32.

12. The Baths of Creuznach. By Ch. Engelmann, M.D.—Heidelberg, 1840. 8vo, pp. 153.

13. Text-book of Anatomy for Students. By A. J. Lizars, M.D., Professor of Anatomy in Mareschal College, Aberdeen. Part II.—Edinburgh, 1842.

14. The Water Cure. A Practical Treatise on the Cure of Diseases by Water, Air, Exercise, and Diet. By James Wilson, M.D. &c.—London, 1842. 8vo, pp. 202. 4s. 6d.

15. The Simple Treatment of Disease deduced from the methods of Expectancy and Revulsion. By J. M. Gully, M.D.—London, 1842. 12mo, pp. 198. 4s. 6d.

16. On Detecting the Presence of Arsenic, particularly in reference to the employment of Marsh's test. By H. H. Watson—Manchester, 1842. 8vo, pp. 27.

17. A Letter to the Right Hon. Lord Francis Egerton, President Elect of the British Association for the Advancement of Science, containing Observations on Statements made by its Officers, &c. By A. Nasmyth, M.R.C.S. F.L.S. F.G.S.—Lond. 1842. 8vo, pp. 58. 2s. 6d.

18. Observations on Life as the Cause of the Vital Phenomena.—London, 1842. 8vo, pp. 16.

19. Remarks on Medical Reform, in a Letter addressed to the Right Hon. Sir James Graham, Bart. By Sir James Clark, Bart. M.D. F.R.S. Physician in Ordinary to the Queen and to the Prince Albert.—London, 1842. 8vo, pp. 30. 1s.

20. Memoir of James Hope, M.D. Physician to St. George's Hospital, &c. By Mrs. Hope. Edited by Klein Grant, M.D. &c.—London, 1842. 8vo, pp. 358. 7s. 6d.

21. Some Remarks on the Education of Medical Students, particularly with reference to those of King's College, London; in a Letter addressed to the Rev. J. Lonsdale, B.D. Principal of the College. By R. B. Todd, M.D., F.R.S., Professor of Pathology, &c. (not published.)—Lond. 1842. 8vo, pp. 32.

22. An Introductory Lecture on Pictorial Anatomy, delivered to the Students of the School of Design, of Edinburgh, and published at the request of the Board. By James Miller, F.R.S.E., &c.—Edinb. 1842. 8vo, pp. 32.

23. Pulmonary Consumption: its Prevention and Cure established, on new views of the Pathology of the Disease. By Henry Gilbert, M.R.C.S.—Lond. 1842. 8vo, pp. 296.

24. The Climate of the South of Devon; and its Influence upon Health: with short accounts of Exeter, Torquay, &c. By Thos. Shapter, M.D., &c.—Lond. 1842. 8vo, pp. 258. 7s. 6d.

25. Animal Chemistry, or Organic Chemistry, in its applications to Physiology and Pathology. By J. Liebig, M.D., &c. Edited by W. Gregory, M.D., &c. Lond. 1842. 8vo, pp. 354. 9s. 6d.

26. The Spas of Homburg, considered with reference to their efficacy in the Treatment of Chronic Disease. By Sir A. M. Downie, M.D.—London, 1842. 12mo, pp. 100. 1s. 6d.

27. An Essay on Diabetes. By H. Bell, D.M.P. Translated by Alfred Markwick.—London, 1842. 8vo, pp. 96.

28. A Treatise on Irritation of the Spinal Nerves, as the Source of Nervousness, Indigestion, &c. &c. By J. E. Riadore, M.D., &c.—London, 1842. 8vo, pp. 306. 5s. 6d.

29. A Practical Treatise on the Diseases of the Scalp. By J. E. Erichsen, Surgeon.—London, 1842. 8vo, pp. 192. (With Plates.) 10s. 6d.

30. Deformities of the Chest, successfully treated. By C. H. Rogers Harrison, Surgeon. Illustrated by Drawings.—Lond. 1842. 8vo, pp. 164. 8s.

31. Commentaries on new doctrines of a dangerous tendency in Medicine, and on the general principles of safe practice. By Sir Alexander Crichton, M.D., F.R.S., &c. &c.—London, 1842. 8vo, pp. 283. 9s.

32. Hastings considered as a Resort for Invalids, &c. By James Mackness, M.D, Physician to the Hastings Dispensary.—London, 1842. 8vo, pp. 151. 4s.

33. A Case of Carcinomatous Stricture of the Rectum, in which the Descending Colon was opened in the Loin. By Alfred Jukes, Surgeon to the General Hospital, Birmingham.—Lond. 1842. 4to, pp. 24, (with Plates.) 3s.

34. The Pharmacopœia of the United States of America. By Authority of the National Convention, held at Washington, A.D. 1840.--Philadelphia, 1842. 8vo, pp. 279.

35. A Bedside Manual of Physical Diagnosis. Second Edit. By Chas. Cowan, M.D. —London, 1842. 12mo, pp. 99. 3s.

36. On the Curative Influence of the Climate of Pau, and the Mineral Waters of the Pyrenees. By A. Taylor, M.D.—Lond. 1842. 8vo, pp. 342.

37. Remarks on Amputation: an Essay, submitted to the Faculty of Physicians of Glasgow, &c. By Alexander King.—Hamilton, 1842. 8vo, pp. 44.

38. A Treatise on Mineral Waters, with particular reference to those prepared at the Royal German Spa, at Brighton. By J. C. A. Franz, M.D.—London, 1842. 8vo, pp. 148. 4s. 6d.

39. On Diseases of the Bladder, and Prostate Gland. With plates. By William Coulson. Third Edition, revised and corrected.—London, 1842. 8vo, pp. 274. 7s.

40. Elements of Chemical Analysis,—Inorganic and Organic. By E. A. Parnell.—London, 1842. 8vo, pp. 309. 10s. 6d.

41. Methodus Medendi; or the Description and Treatment of the Principal Diseases incident to the Human Frame. By Henry M'Cormac, M.D., Professor of Medicine in the Royal Belfast Institution. —London, 1842. 8vo, pp. 574. 16s.

42. On the different Forms of Insanity, in relation to Jurisprudence. By J. C. Prichard, M.D., F.R.S., &c.—Lond. 1842. 8vo, pp. 243. 5s.

43. An Introductory Lecture on the Objects and Nature of Medical Science. By E. Bartlett, M.D., Professor of Medicine in Transylvania University.—Lexington, 1841. 8vo, pp. 18.

44. Observations on Ulcers of the Legs and other Parts, &c. By A. Maxfield, Surgeon to the Hants Infirmary.—London, 1842. 8vo, pp. 80. 5s.

45. A Phrenological and Pathological Inquiry concerning the Physical Characteristics of the Human Teeth and Gums, &c. By C. A. Harris, M.D., &c.—Baltimore, 1841. 8vo, pp. 118.

46. Elements of Physiology, for the use of Students. By Rudolph Wagner, M.D. Part II.—Nutrition and Secretion. Translated from the German, by R. Willis, M.D. —London, 1842. 8vo.

FOREIGN.

1. Solution du Problême de la Population et de la Subsistence. Par Charles Loudon, M.D.—Paris, 1842. 8vo, pp. 329.

2. Nosologia Positiva scritta da Vincenzio Lanza, M.D., Professore nella Cattedra di Medicina pratica della Regia Universita di Napoli, &c. Tomo Primo.—Napoli, 1841. 8vo, pp. 608.

3. Elementa Physiologiæ specialis Corporis Humani. Auctore A. A. Sebastian. Editio altera emendata et aucta—Groningæ, 1842. 8vo, pp. 355.

4. Recherches Anatomiques, Physiologiques, Pathologiques, et Semeiologiques, sur les Glandes Labiales. Par A. A. Sebastian, M.D., &c.—Groningæ, 1842. 4to, pp. 21.

5. De Erysipelate Ambulanti Disquisitio, quam ad summis, &c. Submittet C. E. Fenger, &c.—Havniæ, 1842. 8vo, pp. 208.

6. Erreurs des Médecins, ou Système Chronothermal; traduit de l'Anglois du Dr. Dickson, Par Malvius, A.D.C.—Paris, 1842. 8vo, pp. 580.

7. Die Heilbarkeit des Taubheit. Von Dr. W. Kramer.—Berlin, 1842. 8vo. pp. 56.

### INDEX TO VOL. XIV.

OF THE

### BRITISH AND FOREIGN MEDICAL REVIEW.

| | PAGE |
|---|---|
| Abdomen, on the pendulous . | 215 |
| Abdominal effusion, case of . | 126 |
| Absorption and secretion, physiology of | 564 |
| Addison on the anatomy of the lungs | 39 |
| Agents, medicinal, Liebig on . | 516 |
| Air in the veins . . | 555 |
| Albuminuria, Mr. Robinson on . | 578 |
| Algiers, the fevers of . . | 43 |
| Alkalies, their use in phthisis . | 194 |
| American Pharmacopœia (1842) . | 402 |
| Amputations, statistical account of . | 60 |
| Anatomy, new edition of Soemmering's | 373 |
| general, treatises on . | 478 |
| pictorial, lecture on . | 528 |
| Aneurism of the mesenteric arteries | 64 |
| osseous . . | 581 |
| Angina pharyngea, use of alum in . | 228 |
| Animal kingdom, Dr. Hall on . | 218 |
| Anus, artificial, cases of . | 231, 250-1 |
| Anchylosis, Vrolik on . . | 535 |
| Aortitis, Dr. Chevers on . . | 127 |
| Apoplexia intermeningea . . | 223 |
| Arachnoid, suppuration of . . | 227 |
| Arm presentation, case of . | 582 |
| Arsenic, its use in intermittent fever | 43 |
| mode of detecting . | 258 |
| Arteries, deligation of . . | 21 |
| size of branches and trunks | 574 |
| Artery, axillary, obliteration of . | 254 |
| Atresia vaginæ . . | 563 |
| Asthma, grinders', morbid anatomy of | 580 |
| Auscultation, Dr. Skoda on . | 173 |
| Benzoic acid in gout . . | 52 |
| Bethlem Hospital, pupils at . | 217 |
| Bichat, his discoveries . . | 485 |
| Biographical dictionary, new . | 536 |
| Birth, protracted . . | 256 |
| Bladder, extrophy of . . | 573 |
| Blatin, M., on diseases of women . | 534 |
| Bloodletting, Lisfranc on . | 5 |
| Blood, anatomy, physiology, and pathology of, Mr. Jones on . | 585 |
| Blood-corpuscle, characters of . | 133 |
| globules, Donnè on . | 223 |
| circulation of, in the capillaries | 575 |
| Books for review . . | 601 |
| Boudin, M., on remittent fevers . | 43 |
| Bourgery, M., on the spleen . | 541 |
| lungs . | 546 |
| Bowman, Mr., on the Malpighian bodies . . . . | 567 |
| Brain, softening of . . | 547 |
| Breech case . . . | 256 |
| Bruns, M., his general anatomy | 478, 480 |
| Buffy coat, peculiar state of . | 553 |
| Burns, Lisfranc on . . | 18 |
| Cæsarean section, results of . | 199 |
| Calomel in ophthalmia . . | 559 |
| Calculus, large, in a child . . | 255 |
| Cancer, remarks on . . | 12 |
| Cancer of the lungs . . | 242 |
| penis . . | 563 |
| Carus, Dr., his new cranioscopy . | 65 |
| Cauda equina, tumour of . | 396 |
| Cauterization of the cervix uteri . | 235 |
| Cerebral substance, peculiar affection of | 225 |
| Ceely, Mr., on variolæ vaccinæ . | 397 |
| Cerebro-spinal fluid, treatise on . | 462 |
| Chemistry, animal, Liebig on . | 492 |
| pathological, Dr. Bird on | 576 |
| Chorea, Dr. Babington on . | 134 |
| Chailly, M., on midwifery . | 366 |
| Children's diseases, treatises on . | 203 |
| China, medical missions in . | 216 |
| Chlorosis, M. Boismont on . | 392 |
| Churchill, Dr., on midwifery . | 366 |
| Clark, Sir J., on medical reform . | 296 |
| Cod-liver oil, treatise on . . | 441 |
| Colica pictonum, warm water in . | 61 |
| Collier, Dr., on non-azotized food . | 508 |
| Consumption and marshes . | 44 |
| Consumption, Dr. Campbell on . | 181 |
| Mr. Gilbert on . | 536 |
| Cooper, Mr. B., on capital operations | 132 |
| Copenhagen, health of labourers of | 111 |
| Coronary circulation of the heart . | 575 |
| Corpuscles, the smallest of mammals | 570 |
| the oval of vertebrata | 571 |
| of the nuclei of . | 572 |
| Corpora lutea in med. jurisprudence | 584 |
| Cowan, Dr., his bedside manual . | 536 |
| Cranioscopy, new system of . | 65 |
| Croton oil, new mode of applying . | 229 |
| Cure, proper meaning of the word . | 192 |
| Cysticercus, Mr. Gulliver on . | 49 |
| Death from peas . . . | 576 |
| Delirium tremens, statistics of . | 561 |
| Delivery, spontaneous, mechanism of | 368 |
| Devon, Dr. Shapter on . . | 470 |
| Diabetes, Dr. Bell on . . | 338 |
| mellitus cured (?) . | 246 |
| Diaphragm, ossified . . | 562 |
| Digestion, diseased, mineral waters in | 331 |
| Disease, Liebig's theory of . | 520 |
| Dislocations and fractures, Sir A. Cooper on . . . | 28 |
| Dislocations of the hip-joint . | 34, 37 |
| Dockyard labourers, health of . | 111 |
| Dressing of wounds, Lisfranc on . | 15 |
| Dumbness, curious case of . | 553 |
| Dunglison, Dr. practice of medicine | 220 |
| Ear, pathology of . . | 62 |

PAGE
Education of mothers, Martin on . 527
Electricity in the human body . 562
Endermic method, treatise on . 530
Engel, Dr., on fatty muscle . 543
Entozoa, on the development of . 547
Epistaxis, new mode of treating . 550
Erysipelas, Velpeau on . . 232
Eustachian tube, air douche . 250
Exophthalmos, case of . . 233
Exomphthalmia, case of . . 557
Eyes, hysterical affection of . 246
Fenger, Dr., on the health of labourers 111
Fevers of warm climates . . 43
Fevers, marsh, treatment of . 47
Femur, spontaneous luxation of . 554
Fibrin, on the structure of . . 572
Fingers, reunion of divided . 231
Fistula, intestino-vesical . . 134
thoracic, carious . . 556
Fontana, his discoveries . . 485
Food of animals, Liebig on . 504
Fractures and dislocations, Sir A. Cooper on . . . 28
Fractures, treatment of . . 7
Fracture of end of radius . . 230
of the neck of the thighbone 252
Frankum, Mr., on pendulous belly . 215
Funis, on prolapsus of . . 559
short, case of . . 582
Gangrene from obstruction, treatise on 84
dry, case of . . 502
Gerber, his general anatomy . 479
Gilding surgical instruments . 233
Glanders in man . . . 247
Glands, salivary, extirpation of . 221
Globules in health and disease . 240
Goodsir, Mr., on vegetable organisms 246
on secretion and absorption 564
on the intestinal villi . 566
Gouty concretions, Dr. Ure on . 52
Gout and rheumatism, of mineral waters in . . . 333
Granville, Dr., on mineral waters . 317
Graves, Dr., on cardiac affections . 241
Grew, his discoveries . . 484
Guerin, M., on subcutaneous operations 137
Gullet, muscular fibre of . . 571
Gulliver, Mr., on cysticercus . 49
his notes on Gerber . 478
on the globules of the blood 571
Guy's Hospital Reports . . 122
Guy, Dr., his Hooper's vade mecum 217
Hales, Dr., his discoveries . 485
Hall, Dr. M., on the nervous system 55
his Gulstonian lectures 197
Hare-lip, operation for . . 234
in a child . 250
Hawkins, Mr., on disease of the spine 53
Head, Sir F., on Schlangenbad . 317
Heart, normal dimensions of . 239
affections, Dr. Graves on . 241
signs of valvular disease of . 245
Heat, animal, Liebig on . . 297

PAGE
Hemorrhage after lithotomy . 234
Hemorrhagic diathesis, treatment of 577
case of . 578
Hecker, Dr., on dry gangrene . 84
Henle, Dr., his general anatomy . 478
Hernia, various treatises on . 91
litrica, Riecke on . . 360
after wound . . 556
of the thyroid hole . ib.
radical cure of . . 232-4
use of morphia in . . 581
Hewson, Mr., his discoveries . 486
Hip-joint, spontaneous dislocation of 59
Hooke, Dr., his discoveries . 483
Hooper's Vade Mecum (new edit.) 217
Hope, Dr., memoir of his life . 532
Hughes, Dr., on abdominal effusion 126
malignant dis. of lungs 131
Hydrocephalus . . 205
spontaneous cure of 237
result of puncture in 256
Hydrocephalocele, case of . 246
Hydropathy, or the cold water cure 422
Hygiène, public . . 446
Hysteria in a man . . 246
Indentation of os frontis in labour . 236
Intestinal villi, the structure of . 566
Kayser, Dr., on the Cæsarean section 199
Kidney and bladder conjointly affected 162
Klencke, Dr., his histology . 478-80
Köstlin's microscopic researches . ib.
Labour, new mode of accelerating . 257
Langenbeck, his anatomical plates . 539
Larynx, external fistula of . 232
Lawrence, Mr., on hernia . 91
Ledermüller, his discoveries . 485
Leeches, on the application of . 3
Leeuwenhoeck, his discoveries . 482
Lens, dislocation of . . 252
Leprosy, antiquity of . . 244
Leucoma, new mode of treating . 558
Lieberkuhn, his discoveries . 485
Liebig, Dr., his animal chemistry . 492
Life, observations on . . 521
Lightning, apparent death from . 550
Lisfranc, M., his clinical surgery . 1
Lithotomy, Sir B. Brodie on . 166
Lithotrity, Sir B. Brodie on . 168
cases of . . 249
Lower jaw, congenital luxation of . 238
dislocation of . 252
Lungs, on the anatomy of . 59
malignant disease of . 131
severe wound of . . 255
on the structure of . 546
abscess of, pointing at a distance 578
minute anatomy of . . 573
Luxation, spontaneous, of the femur 554
Lymph-globules of birds . . 571
Magnet, surgical use of . . 557
Majendie on the cerebro-spinal fluid 462
Malgaigne, M., on hernia . 91
Malignant disease of the face . 580

5/

PAGE
Malpighi, his discoveries . . 482
Malpighian bodies, on the structure and use of . . . 567
Marx, Dr., on Paracelsus . 149
Martin on the education of mothers 527
Materia Medica, Dr. Paine on . 213
Medico-chirurgical Transactions . 49
Medical reform, Sir James Clark on 296
Medicine and surgery, relations of . 402
Menstruation, statistics of . 384
pathology of . 391
Metamorphosis of tissue, Liebig on 511
Microscope, results from, in physiology 259
Dr. Mandl on . 478-80
Dr. Bennett on . 478-81
true estimate of . 489
Microscopic researches in physiology 478-80
Midwifery, history of . . 80
practical treatises on . 366
plates on . . 538
Milk, on the fat of . . 222
extemporaneous . . 229
Millipedes discharged from the stomach 572
MINERAL WATERS—
various treatises on . 309-20
general observations on . 310
powers and mode of action of 321
diseases benefited by . 327
*a.* of digestive organs . 331
*b.* gout and rheumatism . 333
*c.* nervous diseases . 336
*d.* paralysis . . 339
*e.* chronic mucous diseases 340
*f.* chronic skin diseases . 341
*g.* scrofula . . 342
*Saline aperient* . 325-342
*a.* Carlsbad (167°) . 342
*b.* Kissingen (cold) . 343
*c.* Marienbad (cold) . 344
*d.* Franzensbad (cold) . 344
*e.* Cheltenham (cold) . 345
*f.* Leamington (cold) . 345
*g.* Scarborough (cold) . 343
*Hot saline* . . 346
*a.* Wiesbaden (160°) . ib.
*b.* Baden-Baden (153°) . ib.
*Hot sulphureous* . . ib.
*a.* Aix-la-chapelle (130°) ib.
*b.* Barèges (130°) . 347
*c.* Bagneres de Luchon (130°) ib.
*Hot alkaline* . . ib.
*a.* Vichy . . ib.
*b.* Mont d'Or . . 350
*c.* Ems (83°–115°) . ib.
*d.* Töplitz (114°–122°) . 351
*e.* Schlangenbad (86°) . ib.
*f.* Wildbad (88°–99°) . ib.
*Simple unmineralized, hot* . ib.
*a.* Pfeffers . . ib.
*b.* Gastein (118°) . 352
*c.* Buxton (82°) . ib.
*d.* Bristol (74°) . ib.
*e.* Matlock (66°) . ib.

PAGE
MINERAL WATERS—
*Simple mineralized, hot.* . 352
*a.* Bath (112°–116°) . 353
*b.* Pyrenees (80°–122°) . ib.
*Chalybeate waters* . . 354
*a.* Spa (cold) . . 355
*b.* Schwalbach (cold) . ib.
*c.* Pyrmont (cold) . ib.
*d.* Marienbad (cold) . ib.
*e.* Bruckenau & Bocklet (cold) ib.
*f.* Tunbridge (cold) . 356
*g.* Niton (cold) . . ib.
*h.* Furnas (hot) . . ib.
*Ioduretted and brominated* . ib.
*a.* Creuznach . . ib.
*b.* Hall . . ib.
*c.* Ischl . . 357
*d.* Woodhall . . ib.
Rules for drinking . . ib.
Dietary during a course of . 358
Missionary medical reports on China 216
Monomania ophthalmica . . 548
Montgomery, Dr., on pregnancy . 207
Motard, M., on state medicine . 446
Mothers, on the education of . 527
Mother's mark, singular case of . 561
Motion, animal, Liebig on . 497
Mucous diseases, mineral waters in . 340
Muscles, new, account of . 376-7
Muscle, fatty, degeneration of . 543
Nasal fossæ, closure of . . 223
Neck of the femur, united fracture of 50
Nerves, M. Valentin on . . 380
Nervous disease, mineral waters in . 336
system, Mr. Mayo on . 525
Nipple, sore, use of catechu in . 582
Nitrate of silver, mode of preserving 557
Nose, enlargement of . . 248
Operations, capital, Mr. Cooper on . 132
Paget, Mr., his report on physiology 259
on the relative size of arteries 574
Palate, on the nerves of . . 222
Panizza's views confirmed . 545
Paracentesis abdominis . . 254
Paracelsus, the real merits of . 149
Paralysis, Dr. Robertson on . 218
mineral waters in . 238
Pelvis, fracture of the, M. Boudin on 39
Penis, cancer of . . . 563
Perforation of stomach, spontaneous 247
Percussion, Dr. Skoda on . 173
new hammer for . 180
Perineum and bladder, Dr. Monro on 540
Peritonitis with effusion . . 549
Pharmacopœia of the United States . 437
Phlebitis, Dr. Silvester's case of . 53
Phrenology, true and false . 65
Phthisis, etiology of . . 225
Physiology and therapeutics, relation of 197
Mr. Paget's report on . 259
Physiological researches . . 56-91
Pin, curious passage of . . 396
Placenta, prolonged retention of . 236

PAGE
Poisoning by arsenic, Mr. Taylor on 122
by sulphuric acid . 584
Polypus of the uterus . . 558
Polypus, uterine, case of . . 583
Practice of medicine, Dr. Dunglison's 220
Pregnancy, Dr. Montgomery on . 207
case of extra-uterine . 560
Preissnitz, account of his water cure 422
Prolapsus of the funis, mode of treating 559
Prostate, enlarged . . 163
Prussian army, revaccination in . 237
Puberty, period of, in negroes . 574
Pulmonary artery, laceration of . 547
Quinine, mode of disguising . 560
Radius, fracture of lower end of . 230
Respiration, cause of the first . 223
Respiration and animal heat, Liebig on 297
Responsibility, legal, treatise on . 529
Rheumatism, Dr. Robertson on . 219
Dr. Greiner on . 412
use of moxa in . 581
and gout, mineral waters in 333
Rhubarb, applied to ulcers . 576
Riecke, Dr. on hernia . . 360
Riadore, Dr. on spinal irritation . 533
Robertson, Dr., on the spine . 219
Rubeola (Rötheln) . . . 206
Saliva, physiology of . . 239
Saw, new cranial . . . 555
Scarlatina, malignant . . 204
Scrofula, mineral waters in . . 342
Secretion and absorption, physiology of 564
peculiar, on the hands . 227
Mr. Goodsir, on . 564
Seton in brain diseases . . 252
Shapter, Dr., on Devonshire . 470
Shaw, Mr. on physiological researches 569
Spina bifida, cure of . . 233
Siebold, M., his history of midwifery 80
Simpson, Dr., on leprosy . . 244
Skin diseases, mineral waters in . 341
Smallpox, congenital . . 235
and vaccination, Dr. Gregory on . . . . . 50
Soemmering, new edit. of his anatomy 373
Spermatozoa, characters of . 572
Spillan, Dr., his therapeutics . 140
Spinal and nervous diseases . 218
Spinal cord, on the anterior column of 222
Spinal cord, function of . . 238
Spinal column, malignant disease of 53
Spinal irritation, treatise on . 533
Spleen, anatomy of . . 541
Sprain, treatment of . 17
Stanley, Mr., luxation of hip-joint . 59
State medicine, treatises on . 446
Stethoscope, its use in fracture . 6
Stomacace . . . . 203
Stomach, muscular coat of . . 546
Stomach, perforation of . . 577
Stomach and colon, displacement of 580
Stone in the bladder, Sir B. Brodie on 165

PAGE
Stricture, Mr. Stafford's treatment of 581
Structural anatomy, history of . 482
Strychnia, tannin an antidote for . 229
Subcutaneous operations, M. Guerin on 137
Suicide, remarkable case of . 257
curious case of . . 583
Surgery, clinical, M. Lisfranc's . 1
Mr. Syme's . . 211
and medicine, relations of 402
Surgical instruments, gilding of . 233
Swammerdam, his discoveries . 482
Syphilis, interesting case of . 578
Tannin, antidote for strychnia . 229
Tetanus, traumatic, case of . 254
Theophrastus (Paracelsus) life of . 147
Therapeutics, manual of . . 140
and physiology, relation of 197
Thomson, respiration and animal heat 297
Thoracic duct, analysis of its contents 573
Thumb, dislocation of . . 231
Tic douloureux from tumour . 549
Tissues, on the pathology of . . 544
Torre, Della, his microscopy . 485
Toynbee, Mr., on the ear . . 62
Tracheotomy, successful case of . 255
Transactions of the prov. association 396
Trachea, rupture of . . 549
Triplets, case of . . . 583
Trusses, hernial, principles of . 101
Tubercles of the lungs, cause of . 184
Tumour, large congenital . 583
Twins, united, case of . . 255
extraordinary case . . 582
Typhus at Rheims . . 224
Typhus abdominalis, diagnosis of . 550
Typhus, pathology of . . 243
Ulcer communicating with the lung 576
Urinary organs, Sir B. Brodie on . 159
Uterus, anterior obliquity of . 370
Uvula, M. Lisfranc on . . 2
Vaccination and smallpox . 50
Vade mecum, the physician's . 217
the anatomist's . 220
Vagina, atresia of . . . 563
Vegetable organisms from the stomach 246
Veins, admission of air into . 555
Venesection, M. Lisfranc on . 21
Verdier, M., on hernia . . 91
Vidal, M., on hernia . . 91
Vital force, Liebig on . . 495
phenomena, cause of . . 521
Vomiting fæcal, prolonged . 579
Vrolik, G., on anchylosis . . 535
Water, the cold, cure . . 422
Waters, mineral, treatises on . 310
Webster, Dr., on lunatic asylums . 217
Walther, M., on medical reform . 402
West, Dr., on puncture of the head 256
Wier Muys, his microscopy . 485
Wilson, Mr. E., his anatomy . 220
Women, mucous diseases of . 534
Wounds, on the dressing of . 15

C. ADLARD, PRINTER, BARTHOLOMEW CLOSE.

